# Use and misuse of common terminology criteria for adverse events in cancer clinical trials

**DOI:** 10.1186/s12885-016-2408-9

**Published:** 2016-07-04

**Authors:** Sheng Zhang, Fei Liang, Ian Tannock

**Affiliations:** Shanghai Cancer Center and Shanghai Medical College, Fudan University, Shanghai, China; Medical Oncology, Princess Margaret Cancer Centre, University of Toronto, Toronto, Canada; Medical Oncology, Shanghai Cancer Center, Fudan University, 270 Dongan Road, 200032 Shanghai, China

**Keywords:** Adverse event, Common terminology criteria, Randomized clinical trial

## Abstract

**Background:**

Common Terminology Criteria for Adverse Events, Version 3.0 (CTCAE v3.0) were released in 2003 and have been used widely to report toxicity in publications or presentations describing cancer clinical trials. Here we evaluate whether guidelines for reporting toxicity are followed in publications reporting randomized clinical trials (RCTs) for cancer.

**Methods:**

Phase III RCTs evaluating systemic cancer therapy published between 2011 and 2013, were reviewed to identify eligible studies, which stated explicitly that CTCAE v3.0 was used to report toxicity. Each AE term and its grade were located in CTCAE v3.0 to determine if they fell within the guidelines provided in the explanatory file.

**Results:**

A total of 166 publications were included in this analysis. Criteria from CTCAE v3.0 were frequently used incorrectly. For example, CATEGORY names such as Metabolic were misreported as AEs in 19 trials, and inappropriate grades for AEs assigned frequently. For example, febrile neutropenia was graded 1 or 2 in 35 of 91 studies (38 %), but the minimum grade for this toxicity is 3. Alopecia was graded 3 or more in 19 of 77 studies (25 %), but the maximum is only grade 2.

**Conclusion:**

The present study provides evidence of poor reporting of toxicity in clinical trials. The study provides a lower estimate for the misuse of AE terms and grades, and implies that other AE terms and grades that conform to CTCAE v3.0 guidelines may have been assigned incorrectly. Inaccurate reporting of toxicity in clinical trials can lead clinicians to make inappropriate treatment decisions.

## Background

Randomized phase III trials (RCTs) are the gold standard in assessing medical interventions. The findings from RCTs enable clinicians to make treatment recommendations, describe the risks and benefits of various treatments, and facilitate shared decision-making [1]. Most cancer therapies have a narrow therapeutic index, and the high levels of toxicity generated by many of them require stringent and uniform standards of reporting to describe the scope and severity of adverse events (AEs). Reproducible and systematic reporting of toxicity allows studies to be more easily compared with one another [2–4] and facilitates the generation of toxicity-related meta-analyses and other secondary analyses [5–7].

The Common Terminology Criteria for Adverse Events (CTCAE) [8] is a uniform system of nomenclature for classifying AEs and their associated severity in cancer clinical trials. It was designed to aid clinicians in the detection and documentation of an array of AEs commonly encountered in oncology. Although CTCAE was designed for use in clinical trials, it is often used in routine care to guide treatment decisions, including drug dosing and supportive care interventions [3, 9]. In 2003, the NCI announced the third revision of the CTC, labeled CTCAE v3.0 [10], which is a comprehensive standardized AE lexicon and grading system for multimodality interventions. The CTCAE v3.0 is the primary method for reporting AEs in medical journals and oncology meetings [8].

The wide use of CTCAE v3.0 has been critical in understanding treatment-related harms and has facilitated comparisons of toxicity profiles among different anticancer reagents and multimodality therapeutics [11, 12]. However, there has been no systematic evaluation of the extent to which reports of phase III RCTs adhere to guidelines associated with CTCAE v3.0 [12] (http://ctep.cancer.gov/protocolDevelopment/electronic_applications/docs/resp_AE_rpt.ppt). The primary aim of the present study was to assess the quality of reporting of AEs in publications describing the results of recent RCTs.

## Methods

### Trial selection

We searched MEDLINE via PubMed (http://www.pubmed.gov) to identify all publications of phase III RCTs assessing systemic therapies for solid tumors published between January 1,2011, and December 31, 2013. The search was performed in April 2014, using the terms “randomized” and “cancer” as keywords. The filters are “subjects = cancer”; “article type” = clinical trial phase III”; “language = English”; “species = humans” and “ages = adult: 18+ years”. Publications were limited to trials exploring pharmacologic interventions in patients with solid tumors. Observational studies, case reports, editorials, letters, meta analyses, publications using pooled data from two or more trials, phase 1 and 2 studies, studies exploring device or behavioral interventions, hematological studies, supportive care studies and studies in which CTCAE v3.0 was not explicitly stated as the toxicity criteria were excluded. If multiple publications were identified from the same trial, the initial publication was used for the analysis.

### Elements of CTCAE v3.0

CTCAE v3.0 was released in 2003 and was followed with a minor revision version. The explanatory PowerPoint file for CTCAE v3.0 entitled ‘Responsible Adverse Event Reporting: Finding Appropriate AE Terms. ’ also accompanied the file (http://ctep.cancer.gov/protocolDevelopment/electronic_applications/docs/resp_AE_rpt.ppt). In CTCAE v3.0, there are twenty-eight CATEGORIES. A CATEGORY is a broad classification of AEs based on anatomy and/or pathophysiology [10]. Within each CATEGORY, AEs are listed accompanied by their descriptions of severity. An AE is a term that is a unique representation of a specific event used for medical documentation and scientific analyses. Grade refers to the severity of the AE. Although generally grades 1 to 5 are available for most AEs, some AEs are listed with fewer than five options for Grade selection.

#### Data extraction

For our study, we reviewed the CTCAE v3.0 file, minor revision file and explanatory file. This process resulted in identifying the 2 key elements: the AE terms and their grades.

Eligible publications were then evaluated for these two elements of CTCAE v3.0. Data extraction was performed independently by two investigators (S.Z and F.L.). Any discrepancy was resolved by consensus among all authors of this study. Cronbach’s alpha was 0.7.

When reviewing the selected publications, each AE term (or its obvious synonym) and its grade were located in the pdf file of CTCAE v3.0 and its revision with the ‘search’ tool as instructed by the guideline (http://ctep.cancer.gov/protocolDevelopment/electronic_applications/docs/resp_AE_rpt.ppt). If it does not fit an allowed pattern, then it is regarded “misuse”.

The AE terms/grades could be described in the text of the article, or summarized in tables or supplemental documents. Since the most important AE terms/grades are summarized most often in tables, usually with corresponding grades, we evaluated the content of AE tables and their standardization across studies. If AE tables were shown in an online appendix rather than in the main paper, the online documents were also analyzed.

Additional data extracted from each trial included funding, the study sample size, intervention type, use of placebo control, cancer type, cancer stage, publication year, journal name, impact factor and whether primary endpoint was met.

#### Statistical analysis

Results of the analysis were summarized by descriptive statistics.

## Results

### Characteristics of selected RCTs

**Fig 1:**
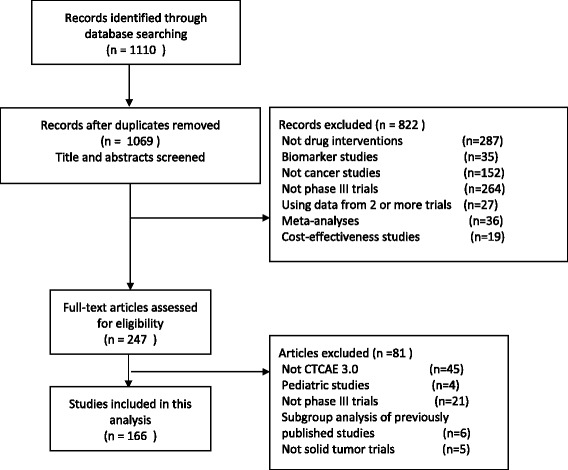
Flowchart of screening of randomized clinical trials included in this analysis

From 1110 articles screened initially, 166 publications describing RCTs were included in the present analysis. The selection process and reasons for exclusion are shown in Fig. 1.

The characteristics of the included publications included are listed in Table 1. These 166 publications reported data on 139,932 patients (median, 836; range, 154–4,984). The most common tumor type explored was lung cancer (25 %), and chemotherapy plus targeted therapy was the most common intervention (38 %). Most trials (87 %) were funded at least in part by industry. Forty-three percent of the trials were positive based on the stated primary outcomes. Seventy-seven percent of articles were published in two journals (*Journal of Clinical Oncology*; and *Lancet Oncology*; Table 1). Eighty-eight percent of papers included one table describing AEs and 9 % had two AE tables in the main paper. Three percent of articles showed the AE tables only in the online appendix.

**Table 1:** Trial Characteristics (*N* = 166)

Characteristic	No.	%
Sample size		
Median	836	
Range	154-4,984	
Placebo controlled	68	41
Intervention type		
Chemotherapy	42	25
Targeted therapy	61	37
Chemotherapy plus targeted therapy	63	38
Trial met the primary end point	71	43
Funding source		
Industry	111	67
Government	10	6
Industry and government	33	20
Not reported	12	7
Cancer type		
Breast	38	23
Colorectal	17	10
Lung	41	25
Gastric or Gastroesophageal	10	6
Other	59	35
Journal		
Annals of Oncology	12	7
The New England Journal of Medicine	10	6
Journal of Clinical Oncology	76	45
Lancet Oncology	50	30
Other	18	11
Year of publication		
2011	49	30
2012	53	32
2013	64	38
Impact factor of journals		
Median	19.6	
Range	3-53	
Cancer stage		
Adjuvant and/or neoadjuvant	25	15
Metastatic	141	85

### Reporting of adverse events

The reporting of toxicity in the publications was often restricted to severe AEs (30 %) and/or frequent AEs (64 %). Most studies pooled AEs of varying severity (89 %). The evaluation of the AE descriptors and their grades was based on data provided by these tables.

Standardized descriptive terms for AEs are required by CTCAE v3.0. However, heterogeneous and non-standardized AE terms were used widely in the publications. For example, Anemia (Hemoglobin should be used), Neutropenia (Neutrophils should be used), Thrombocytopenia (Platelets should be used) were frequent descriptive terms. In the 155 studies where these AEs were included, only 2 % used the correct form. The other examples were shown in Table 2. However, this kind of “misuses” does not impact on the ability of a reader to understand the toxicity profile of the interventions being studied, and was regarded as clinically insignificant by the consensus of our team.

**Table 2:** Examples of Frequent/Representative Non-standardized Terms according to CTCAE v3.0

Category	Descriptors in the Articles	Correct Form or Comments	Frequency
Blood and lymphatic	Anemia Neutropenia Thrombocytopenia	Hemoglobin Neutrophils Platelets	133/146 147/151 104/134
Constitutional	Edema Thromboembolic events Fatigue; asthenia Deterioration in general physical condition	Should be Edema-limb or similar Not an AE term Should use fatigue; they are separate terms in CTCAE v4.0. Not an AE term	54/67 69 14 19
	Decreased appetite		23/49
gastrointestinal	Pyrexia Yellow skin Nausea-vomiting Lacrimation Nasopharyngitis Paresthesia Azotemia	Should use anorexia Not an AE term Not an AE term Not an AE term Not an AE term Not an AE term Not an AE term	16 11 21 29 8 18 14
Other	Thyroid disorders Neutropenic fever Glossodynia Dysphonia Abdominal distention Renal impairment Menopausal symptoms Skin exfoliation Jaundice Psychiatric disorders Epistaxis Mucosal inflammation	Not an AE term Not an AE term Not an AE term Not an AE term Not an AE term Not an AE term Not an AE term Not an AE term Not an AE term Not an AE term Not an AE term Not an AE term	9 22 31 8 15 12 21 29 16 18 9 23

Abbreviations: *AE* adverse event, *CTC* common terminology criteria for adverse events

A CATEGORY is not an AE and should not be reported alone. However, the CATEGORY names such as Constitutional symptoms, Cardiac general, Metabolic, Vascular and others were reported in 19 articles as AEs (Table 3). This type of misuse is discouraged in the explanatory file of CTCAE v3.0, because it does not provide useful and precise information about the toxicity profile.

**Table 3:** Examples of Misuse of CTCAE v3.0 with Clinical Relevance (*N* = 166)

Section	Descriptors in the Articles	Correct Form/Comment	Frequency
AE terms	Constitutional symptoms Cardiac general Metabolic Hemorrhage	Not AE terms; Category names cannot be reported as AEs	19 18 14 19
Grades	Febrile neutropenia grade 1 or 2 Alopecia grade 3 Dysgeusia grade 3 Dyspepsia grade 4 Hyperpigmentation grade 3 Pruritus grade 4 Renal failure grade 1 or 2 Cough grade 4 Hot flash grade 4 Libido grade 3	At least grade 3 Maximum grade 2 Maximum grade 2 Maximum grade 3 Maximum grade 2 Maximum grade 3 At least grade 3 Maximum grade 3 Maximum grade 3 Maximum grade 2	35/91 19/77 7/23 6/26 5/19 3/18 8/29 4/81 6/33 4/28

Abbreviations: *AE* adverse event, *CTC* common terminology criteria for adverse events. Note: Detailed information is described in the Result section

Misreporting of grades of AEs was detected in 47 % of the publications, and this is likely to be a substantial underestimate. Febrile neutropenia was graded 1 or 2 in 35 of 91 papers (38 %), but the minimum grade for this term in CTCAE v3.0 is 3. Alopecia was graded 3 or more in 19 or 77 studies (25 %), whereas grade 2 is the maximum for this term. Other examples of inappropriate grading as well as their detected frequency in the publications are given in Table 3.

## Discussion

A careful balance between efficacy and toxicity is of primary importance in medical interventions. Concerns have been raised previously that anticancer drugs have toxicities that might outweigh their benefits [11]. AE reporting is a critical component in the conduct and evaluation of clinical trials [13]. With approximately 1,000 standardized descriptive terms, CTCAE v3.0 has become the worldwide standard dictionary for reporting AEs in cancer clinical trials [8]. To our knowledge, this is the first large-scale study evaluating the conformity of oncology RCTs publications using CTCAE v3.0 to the corresponding guideline.

Our study provides evidence of poor reporting of toxicity in clinical trials.

Overall, many articles included had some deficiencies or incorrect reporting of AE terms and grades with possible clinical relevance. Concerning that many publications only reported the “pooled,” “selected,” or “worst” AEs which cannot allow for detailed analysis and that we only evaluated the AEs in the tables, the actual number of misused AE terms and grades maybe even higher. In addition, without the access to individual toxicity data, our analysis was only based on the reported toxicity data in trials. This suggests that the undetectable and inaccurate grades of other AE terms may also exist.

It was reported that the subjective AE such as fatigue might be variable when they were assessed by different health practitioners [14]. The objective AEs are generally more consistent and accurate when they are supported by laboratory or imaging results [8]. However, it was demonstrated even for this kind of high-fidelity objective AEs, there are considerable inconsistencies between clinical trial adverse events entered into the Clinical Data Update System, the NCI’s electronic database, and in subsequent publications [15]. Our results further extended these findings, specifically evaluating the quality of reporting toxicity in the context of CTCAE v3.0.

There is one potential reason for the observed problems in our analysis. A lack of authors’ awareness of the explanatory file/guideline for CTCAE 3.0 is a likely contributing factor. It is possible that some authors are not familiar with this document compromising the correct use of CTCAE v3.0.

There are some potential limitations in our study. We restricted our analysis to randomized phase III trial publications for solid tumor treatments in recent years, although adherence to CTCAE v3.0 in phase II trials, hematologic malignancy trials and trials testing multimodality treatment (for example, radiation therapy) should also be required. Moreover, CTCAE v4.0 was released in 2009 and it was gradually implemented recently. Because oncology studies usually take years to complete, only a few publications of RCTs report toxicity with this new version currently. However, the essential parts of CTCAE (AE terms and grades) remain similar. It is possible the problems identified in this analysis would carry over to 4.0 and to future versions, so they need to be recognized and corrected.

## Conclusion

Our study provides a lower estimate for reporting toxicity in the context of CTCAE guideline. Inaccurate reporting of toxicity can lead clinicians to make inappropriate decisions.

## Abbreviations

CTCAE v3.0, common terminology criteria for adverse events; NCI, national cancer institute; RCT, randomized clinical trials
